# Beyond traditional—the sick cirrhosis patient scores integrating cytokine and immune profiling for precision prognosis in hospitalized cirrhosis patients may help predict infection, ICU admission and long-term death post hospitalization

**DOI:** 10.3389/fmed.2026.1736956

**Published:** 2026-01-21

**Authors:** Cyriac Abby Philips, Tharun Tom Oommen, Arif Hussain Theruvath, Aryalakshmi Sreemohan, Ambily Baby, Ansu Abu Alex, Sunitha Thomas, Sunitha Mary John, Rizwan Ahamed, Ajit Tharakan, Philip Augustine

**Affiliations:** 1 Department of Clinical and Translational Hepatology, The Liver Institute, Center of Excellence in Gastrointestinal Sciences, Rajagiri Hospital, Kochi, Kerala, India; 2 Clinical Research Division, The Liver Institute, Center of Excellence in Gastrointestinal Sciences, Rajagiri Hospital, Kochi, Kerala, India; 3 Department of Gastroenterology and Advanced GI Endoscopy, Center of Excellence in Gastrointestinal Sciences, Rajagiri Hospital, Kochi, Kerala, India; 4 Department of Advanced Flow Cytometry Immunophenotyping Facility, Rajagiri Hospital, Kochi, Kerala, India; 5 Department of Pathology and Laboratory Services, Rajagiri Hospital, Kochi, Kerala, India; 6 Department of Biochemistry, Rajagiri Hospital, Kochi, Kerala, India

**Keywords:** ascites, decompensated cirrhosis, encephalopathy, MELD, portal hypertension, sepsis

## Abstract

**Introduction:**

Inflammatory biomarkers and immune cell function may provide prognostic value beyond traditional severity scores in hospitalized cirrhosis patients. We aimed to characterize inflammatory and immune profiles to determine their predictive value for ICU admission, infection, and long-term mortality, and to develop novel scoring systems.

**Patients and methods:**

This retrospective observational cohort study enrolled 78 hospitalized cirrhosis patients. Comprehensive inflammatory profiling included 12 cytokines (multiplex immunoassay), flow cytometry immune markers, and acute phase reactants (e.g., procalcitonin). Outcome measures included ICU admission, infection, and death within 12–24 months. Multivariable logistic regression was used to identify independent predictors and develop three outcome-specific scoring systems.

**Results:**

The cohort had a mean MELD3 of 25.14; 38.5% required ICU admission, and 12–24-month mortality was 43.6%. Independent predictors for infection were procalcitonin (≥0.40 ng/mL), IL-6 (≥84 pg./mL), and creatinine (≥1.35 mg/dL) (AUC 0.74). ICU admission was predicted by hepatic encephalopathy, variceal bleeding, procalcitonin (≥0.40 ng/mL), and IL-6 (≥53.69 pg./mL) (AUC 0.82). Long-term mortality was predicted by ICU admission, hemoglobin (≤11.5 g/dL), EGF (≤2.9 pg./mL), and absolute nucleated cell count (≤3,600/μL) (AUC 0.821).

**Conclusion:**

Biomarkers reflecting systemic inflammation (IL-6, procalcitonin), immune paresis (low nucleated cell count), and failed regenerative capacity (low EGF) strongly predicted adverse outcomes. Integrating these markers into the proposed ‘Sick Cirrhosis Patient Scores’ may improve risk stratification, but these models require rigorous external validation.

## Introduction

Cirrhosis represents a significant global health burden, with increasing prevalence driven by metabolic dysfunction-associated steatotic liver disease (MASLD) and alcohol-related liver disease (1, 2). Despite advances in management, hospitalized patients with cirrhosis face substantial morbidity and mortality, with intensive care unit (ICU) admission rates ranging from 20–40% and ICU mortality ranging from 34 to 72% and in-hospital mortality up to 80% in decompensated disease (3, 4). Current prognostic models, including the Model for End-Stage Liver Disease-3 (MELD3) and Child-Turcotte-Pugh (CTP) scores, primarily rely on markers of hepatic synthetic function and end-organ dysfunction but inadequately capture the inflammatory milieu that drives disease progression, infections and acute or unstable decompensations (5, 6).

The pathophysiology of cirrhosis involves complex interactions between hepatic dysfunction, portal hypertension, and systemic inflammation. Bacterial translocation from the gut, impaired hepatic clearance of pathogen-associated molecular patterns (PAMPs), and damage-associated molecular patterns (DAMPs) perpetuate a state of chronic inflammation (7–9). This inflammatory state, characterized by elevated pro-inflammatory cytokines including interleukin-6 (IL-6), tumor necrosis factor-alpha (TNF-*α*), and the interleukin-1 family, contributes to repeated hospitalizations, organ failures, development of acute-on-chronic liver failure (ACLF), and increased mortality (10, 11).

Recent evidence suggests that inflammatory biomarkers may provide incremental prognostic value beyond traditional scoring systems (12–14). However, comprehensive inflammatory profiling using multiplex cytokine analysis combined with immune cell markers has not been systematically evaluated in hospitalized cirrhosis patients. Furthermore, the temporal dynamics of inflammatory markers and their differential associations with early versus late outcomes remain poorly characterized. In this context, the concept of precision medicine in hepatology necessitates moving beyond one-size-fits-all approaches to risk stratification. Identifying distinct inflammatory phenotypes could enable targeted therapeutic interventions and optimized resource allocation.

This study aimed to comprehensively characterize the inflammatory cytokines and immune profile of hospitalized cirrhosis patients and determine the independent predictive value of inflammatory biomarkers for ICU admission, infection and mortality. We hypothesized that: (1) specific inflammatory markers would independently predict adverse outcomes beyond traditional clinical variables and that (2) a simple yet novel scoring system incorporating inflammatory and immune markers could outperform existing prognostic tools. This study was approved by the Rajagiri Hospital Institutional Ethics Committee (approval obtained prior to data collection). Given the retrospective nature of the study, which utilized only routinely collected clinical data and residual samples obtained as by-products of standard clinical care, the ethics committee granted a waiver of written informed consent in accordance with Indian Council of Medical Research (ICMR) National Ethical Guidelines for Biomedical and Health Research (2017). These guidelines permit the use of anonymized residual clinical samples for research with ethics committee approval when individual consent is impracticable. All data were de-identified prior to analysis, and patient confidentiality was maintained throughout the study. The study was conducted in accordance with the Declaration of Helsinki and local institutional requirements.

## Patients and methods

### Study design and population

This retrospective observational cohort study was designed to comprehensively evaluate inflammatory cytokine and immune cell biomarkers in hospitalized cirrhosis patients and their association with clinical outcomes. The study was conducted at a tertiary care private teaching institute, the Rajagiri Hospital, at Aluva, Kerala, India. The study enrolled 78 patients with confirmed cirrhosis who were hospitalized for cirrhosis-related complications, with follow-up extending between 12–24 months to capture long-term mortality outcomes. Inclusion criteria required patients to be at least 18 years of age with a confirmed diagnosis of cirrhosis established either histologically or through combined clinical and radiological criteria. All participants needed to be hospitalized for cirrhosis-related complications and have complete inflammatory biomarker measurements available for analysis. The study excluded patients with active malignancy other than hepatocellular carcinoma, HIV infection, those receiving immunosuppressive therapy, and any patients without biomarker panels, ensuring a homogeneous population without confounding immunological conditions.

### Outcome definitions

Three primary outcomes were evaluated. Infection at hospitalization was defined as documented bacterial infection present at the time of index hospital admission, diagnosed according to established criteria. Infections included spontaneous bacterial peritonitis (culture-positive or culture-negative with ascitic fluid polymorphonuclear cell count ≥250/mm^3^), urinary tract infection (symptomatic with positive urine culture), pneumonia (clinical symptoms with radiographic confirmation), bloodstream infection (positive blood culture with clinical signs of systemic infection), skin and soft tissue infections, and *Clostridioides difficile* infection. ICU admission was defined as transfer to the intensive care unit at any point during the index hospitalization. Patients admitted directly to the ICU were classified as ICU admissions. Long-term mortality was defined as all-cause death occurring between 12 and 24 months following the index hospitalization, ascertained through medical records review and telephone follow-up.

### Laboratory methods

#### Sample collection and pre-analytical handling

Blood samples for inflammatory biomarker profiling were collected within 24 h of hospital admission, typically concurrent with routine admission laboratory testing. For patients presenting with acute variceal bleeding, samples were obtained during emergent hemodynamic stabilization but before or concurrent with endoscopic intervention. Samples were collected prior to or within 6 h of empiric antibiotic initiation when applicable. Blood was collected in serum separator tubes without anticoagulant, allowed to clot for 30 min at room temperature, and centrifuged at 3000 rpm for 10 min within 2 h of collection. Serum was aliquoted and stored at −80 °C until batch analysis. Samples underwent a single freeze–thaw cycle for analysis. The timing of sample collection relative to interventions was not fully standardized, which is acknowledged as a limitation inherent to retrospective biomarker studies.

#### Inflammatory marker assessments

The inflammatory profiling employed state-of-the-art multiplex immunoassay technology using the Randox Cytokine Array Platform (Evidence Investigator, Randox Laboratories, United Kingdom) to simultaneously measure 12 cytokines including interleukins (IL-1*α*, IL-1β, IL-2, IL-4, IL-6, IL-8, IL-10), interferon-gamma (IFN-*γ*), and tumor necrosis factor-alpha (TNF-α). Additionally, growth factors including epidermal growth factor (EGF) and vascular endothelial growth factor (VEGF), along with the chemokine monocyte chemoattractant protein-1 (MCP-1), were quantified. Rigorous quality control measures were implemented with intra-assay coefficients of variation maintained below 10% and inter-assay coefficients below 15%, ensuring high reproducibility and reliability of measurements.

#### Immune cell surface markers analysis

These were analyzed using flow cytometry (Beckman Coulter, Inc., California, United States) to quantify neutrophil CD64 (nCD64), monocyte HLA-DR (mHLA-DR), and monocyte CD14 (mCD14) expression levels, reported as mean fluorescence intensity (MFI). The Sepsis Index was calculated as a derived parameter from these flow cytometric measurements based on previous standardized descriptions. Acute phase reactants were assessed using established methods, with procalcitonin measured by chemiluminescence assay and C-reactive protein quantified through immunoturbidimetry. This comprehensive panel provided both pro-inflammatory and anti-inflammatory marker assessment, enabling detailed characterization of the inflammatory milieu in cirrhotic patients.

#### Blinding

Outcome assessment was performed retrospectively through medical records review by investigators blinded to biomarker results. Biomarker assays were performed in batch by laboratory personnel blinded to clinical outcomes.

## Statistical analysis framework

Statistical analyses were conducted using R (version 4.3.0; R Foundation for Statistical Computing, Vienna, Austria) and MedCalc Software (version 23.3.5; Ostend, Belgium). Claude Opus 4.1 (Anthropic, San Francisco, USA) was used to prepare tables.

### Descriptive and univariate analyses

Comprehensive descriptive statistics were calculated for each variable within comparison groups, including mean ± standard deviation (SD) using Bessel’s correction for unbiased variance estimation, standard error, and median with interquartile range (IQR, 25th-75th percentile using linear interpolation when necessary). Descriptive statistics and univariate analyses were conducted using all available data for each variable (available case analysis). Results were presented as mean ± SD for parametric data and median (IQR) for non-parametric data. Univariate comparisons between groups employed Student’s t-test for normally distributed continuous variables, with Mann–Whitney U test utilized for non-parametric data. Categorical variables were compared using chi-square tests or Fisher’s exact tests when expected cell counts were below five. Effect sizes were calculated using Cohen’s d for continuous outcomes and odds ratios for categorical variables, providing standardized measures of association strength independent of sample size.

### Candidate predictor variables

The following variables were considered as candidate predictors in model development: demographic variables (age, sex); clinical variables (etiology of cirrhosis, hepatic encephalopathy, acute variceal bleeding, hepatocellular carcinoma, infection status, acute kidney injury, hyponatremia, hypokalemia, septic shock, acute-on-chronic liver failure); traditional severity scores (MELD3, Child-Turcotte-Pugh score); inflammatory biomarkers from the multiplex panel (IL-1*α*, IL-1β, IL-2, IL-4, IL-6, IL-8, IL-10, IFN-*γ*, TNF-α, EGF, VEGF, MCP-1); flow cytometry immune markers (nCD64, mHLA-DR, mCD14, Sepsis Index); acute phase reactants (procalcitonin, C-reactive protein); and standard laboratory parameters (hemoglobin, white blood cell count, platelet count, total bilirubin, albumin, creatinine, INR, sodium, potassium). Traditional severity scores (MELD3, CTP) were allowed to enter and exit models during backward selection without being forced into final models.

### Multivariate modeling

Variables with *p* < 0.05 in univariable analysis were considered for inclusion in the multivariable logistic regression model. To avoid multicollinearity, when two variables were highly correlated (r > 0.80), only the variable with stronger univariable association was retained. Multivariate logistic regression models were developed using backward stepwise selection procedures, with *p* < 0.05 required for variable entry and *p* > 0.10 for removal, balancing model parsimony with explanatory power. For the multivariable logistic regression models, a complete case analysis was performed; patients with missing data for any predictor variable within a given model were excluded from that specific analysis, as noted in the results. When distributions were skewed, these were log-transformed before entering regression models. Variable selection incorporated both statistical significance and clinical relevance, ensuring models remained interpretable and clinically applicable. For continuous variables showing significant association (*p* < 0.05), receiver operating characteristic (ROC) curves were constructed to determine optimal cut-points. The Youden index (sensitivity + specificity – 1) was maximized to identify the optimal threshold for each biomarker. Area under the curve (AUC) with 95% confidence intervals was calculated to assess discriminatory ability. The final model was developed using backward stepwise selection with *p* < 0.05 for retention. Adjusted odds ratios (OR) with 95% confidence intervals (CI) were calculated for each predictor. Based on regression coefficients and clinical interpretability, a point-based risk scoring systems were developed. Model performance was assessed through the Nagelkerke R^2^ for logistic models and the concordance index (C-statistic, equivalent to AUC for binary outcomes) while clinical utility was assessed via sensitivity, specificity, positive predictive value (PPV), and negative predictive value (NPV) at various cut-points.

### Missing data handling

Complete case analysis was used for multivariable modeling. Missing data were primarily attributable to procalcitonin (18 patients, 23.1%) and flow cytometry markers including nCD64, mHLA-DR, mCD14, and Sepsis Index (9 patients, 11.5%), reflecting samples not collected rather than values below detection limits. Multiple imputation was not performed given the mechanism of missingness and the limited sample size. Sensitivity analyses comparing baseline characteristics and outcomes between patients with complete versus incomplete data were performed to assess potential selection bias.

### Score development methodology

To complement the dichotomized scoring systems and assess potential information loss from categorization, univariable logistic regression was performed with biomarkers modeled as continuous variables. Odds ratios were calculated per one standard deviation increase to allow comparison of effect sizes across biomarkers with different units. Continuous biomarker variables with right-skewed distributions (IL-6, procalcitonin, EGF) were log-transformed prior to regression modeling to satisfy linearity assumptions. For variables retained in final multivariable models, receiver operating characteristic (ROC) curve analysis was performed to identify pragmatic clinical thresholds. Cut-off values were determined by maximizing the Youden index (J = sensitivity + specificity − 1) using the same derivation dataset, which may optimize apparent rather than external performance. Point assignments for scoring systems were derived from the relative magnitude of regression coefficients. Variables with larger adjusted odds ratios (indicating stronger association with the outcome) were assigned proportionally more points. Specifically, the ratio of each variable’s coefficient to the smallest coefficient in the model was calculated and rounded to the nearest integer, with scaling applied to produce clinically practical point values (range 1–5 points per variable). No post-hoc recalibration was performed. These cut-points and point assignments should be considered exploratory and require external validation before clinical application.

### Internal validation

To assess potential overfitting and estimate optimism in model performance, repeated stratified k-fold cross-validation was performed for each scoring system. This approach, which provides more stable estimates than single cross-validation, used 10 repetitions of 5-fold stratified cross-validation (50 total iterations). For each iteration, the dataset was randomly partitioned into five folds while maintaining the proportion of outcome events, with models iteratively trained on four folds and validated on the held-out fold. Cross-validated AUC was reported as the mean across all 50 iterations with 95% confidence intervals derived from the 2.5th and 97.5th percentiles of the distribution. Optimism was calculated as the difference between apparent (full-sample) and cross-validated AUC.

## Results

### Patient characteristics and baseline demographics

The study cohort comprised 78 hospitalized patients with cirrhosis, with a mean age of 58.74 ± 9.72 years and a marked male predominance (87.2%). The predominant etiology was metabolic dysfunction-associated steatotic liver disease (MASLD), accounting for 55.1% of cases, followed by alcohol-related cirrhosis in 38.5% of patients. The cohort demonstrated significant disease severity, with a mean MELD3 score of 25.14 ± 7.34 and Child-Turcotte-Pugh score of 8.87 ± 1.58, indicating predominantly Child B and C cirrhosis. Clinical complications were prevalent throughout the cohort. Primary indications for hospitalization included ascites requiring management (45 patients, 57.7%), documented infection or sepsis (21 patients, 26.9%), acute-on-chronic liver failure (15 patients, 19.2%), acute variceal bleeding (13 patients, 16.7%), and hepatic encephalopathy (10 patients, 12.8%), with some patients presenting with overlapping indications. Among the 15 patients meeting criteria for ACLF (19.2%), disease severity was distributed as follows: ACLF Grade 1 (single organ failure) in 9 patients (60%), Grade 2 (two organ failures) in 4 patients (26.7%), and Grade 3 (three or more organ failures) in 2 patients (13.3%), classified according to EASL-CLIF Consortium criteria. No patients in this cohort underwent liver transplantation during the follow-up period, reflecting limited transplant access in our setting. The ICU admission occurred in 30 patients (38.5%), with in-hospital mortality remaining low at 2.6%, while 12–24-month mortality was substantially higher at 43.6% (Supplementary Table 1).

Comprehensive inflammatory profiling revealed significant elevations across multiple cytokine families. IL-6 demonstrated the highest absolute levels (median 98.73 pg./mL, IQR: 47.22–190.79), with 52.6% of patients exceeding normal ranges. TNF-*α* was elevated in 48.7% of patients (median 18.13 pg./mL), while IL-1β elevation occurred in 41.0%. The anti-inflammatory cytokine IL-10 was elevated in 29.5% of patients with mean levels of 10.55 ± 27.61 pg./mL. Among chemokines, MCP-1 showed marked elevation (median 284.00 pg./mL) in 55.1% of patients. To contextualize the magnitude of inflammatory dysregulation, biomarker levels were compared to published reference ranges for healthy adults. IL-6 levels in our cohort (median 98.73 pg./mL) were markedly elevated compared to healthy reference values (<7 pg./mL), representing greater than 14-fold elevation. Procalcitonin levels (median 0.40 ng/mL) exceeded normal thresholds (<0.1 ng/mL). Notably, EGF levels were substantially reduced compared to healthy adult ranges (typically 200–600 pg./mL), with our cohort demonstrating a median of only 10.47 pg./mL, suggesting impaired regenerative capacity in this hospitalized cirrhosis population. Flow cytometry-based immune cell markers revealed elevated nCD64 expression in 36.2% and elevated Sepsis Index in 31.9% of patients (Supplementary Table 2).

### Predictors of infection

**Figure 1:**
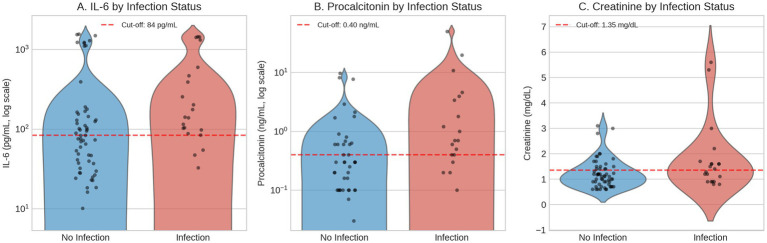
Biomarker distributions by infection status at hospitalization. Violin plots with individual data points showing **(A)** interleukin-6 (IL-6), **(B)** procalcitonin, and **(C)** serum creatinine stratified by presence (*n* = 21) or absence (*n* = 57) of infection. Procalcitonin data available for 60 patients. Y-axes for IL-6 and procalcitonin displayed on logarithmic scale. Dashed red lines indicate ROC-derived cut-off values used in the Sick Cirrhosis Patient Score Part A (IL-6 ≥ 84 pg./mL; procalcitonin ≥0.40 ng/mL; creatinine ≥1.35 mg/dL).

Among 78 hospitalized patients with cirrhosis, 21 (26.9%) had infection. Univariable analysis was performed on all 78 patients (21 with infection, 57 without). Multivariable logistic regression analysis was conducted on 60 patients with complete biomarker data (19 with infection, 41 without). Eighteen patients (23%) were excluded from multivariable analysis due to missing values in one or more predictor variables. Univariable analysis (Supplementary Table 3) identified eight significant predictors of infection (*p* < 0.05), with procalcitonin demonstrating the strongest association (median 0.70 vs. 0.30 ng/mL, *p* = 0.005), followed by interleukin-6 (median 142.13 vs. 83.11 pg./mL, *p* = 0.008) and tumor necrosis factor-alpha (median 23.42 vs. 13.30 pg./mL, *p* = 0.033). Multivariable logistic regression analysis of 60 patients with complete data identified three independent predictors of infection in hospitalized cirrhosis patients: procalcitonin (AUC 0.729; adjusted OR 2.81, 95% CI: 1.42–5.55, *p* = 0.003), interleukin-6 (AUC 0.707; adjusted OR 2.10, 95% CI: 1.10–4.01, *p* = 0.024), and serum creatinine (AUC 0.634; adjusted OR 1.89, 95% CI: 1.01–3.55, *p* = 0.048). Based on ROC-derived optimal cut-offs, we developed a simplified 5-point scoring system (The Sick Cirrhosis Patient Score Part A for Infection at Hospitalization, Table 1; Figure 1): procalcitonin ≥0.40 ng/mL (2 points), IL-6 ≥ 84 pg./mL (2 points), and creatinine ≥1.35 mg/dL (1 point). This scoring system effectively stratified patients into three risk categories with significantly different infection rates: low risk (0–1 points, 4.5% infection rate, *n* = 22), moderate risk (2–3 points, 27.3% infection rate, *n* = 22), and high risk (4–5 points, 56.3% infection rate, *n* = 16; *p* < 0.001 for trend). The combined model had an AUC of 0.74. Using a cut-off of ≥3 points provided balanced performance with approximately 68% sensitivity and 68% specificity. For screening purposes, a cut-off of ≥2 points increased sensitivity to approximately 79% with a negative predictive value of 83%, while a cut-off of ≥4 points optimized specificity to approximately 85%. When modeled as continuous variables, the key biomarkers retained significant associations with their respective outcomes. For infection, each standard deviation increase in log-transformed IL-6 was associated with 75% higher odds of infection (OR 1.75, 95% CI: 1.05–2.90, *p* = 0.030), while log-transformed procalcitonin showed the strongest continuous association (OR 2.41 per SD, 95% CI: 1.25–4.65, *p* = 0.009). Internal validation using repeated stratified k-fold cross-validation (10 repeats × 5 folds) yielded a cross-validated AUC of 0.749 (95% CI: 0.354–1.000) compared to the apparent AUC of 0.759, indicating minimal optimism of 0.009 AUC units. The wide confidence interval reflects the inherent variability associated with the limited sample size and event count.

**Table 1:** The Sick Cirrhosis Patient Score Part A for infection at hospitalization.

Parameter	Cut-off value	Points
IL-6	≥ 84 pg./mL	2
Procalcitonin	≥ 0.40 ng/mL	2
Creatinine	≥ 1.35 mg/dL	1
Total score		0 to 5

Risk stratification	
Low risk (0–1 points)	~ 5% infection rate
Moderate risk (2–3 points)	~ 27% infection rate
High risk (4–5 points)	~ 56% infection rate

IL, interleukin.

### Predictors of ICU admission

**Figure 2:**
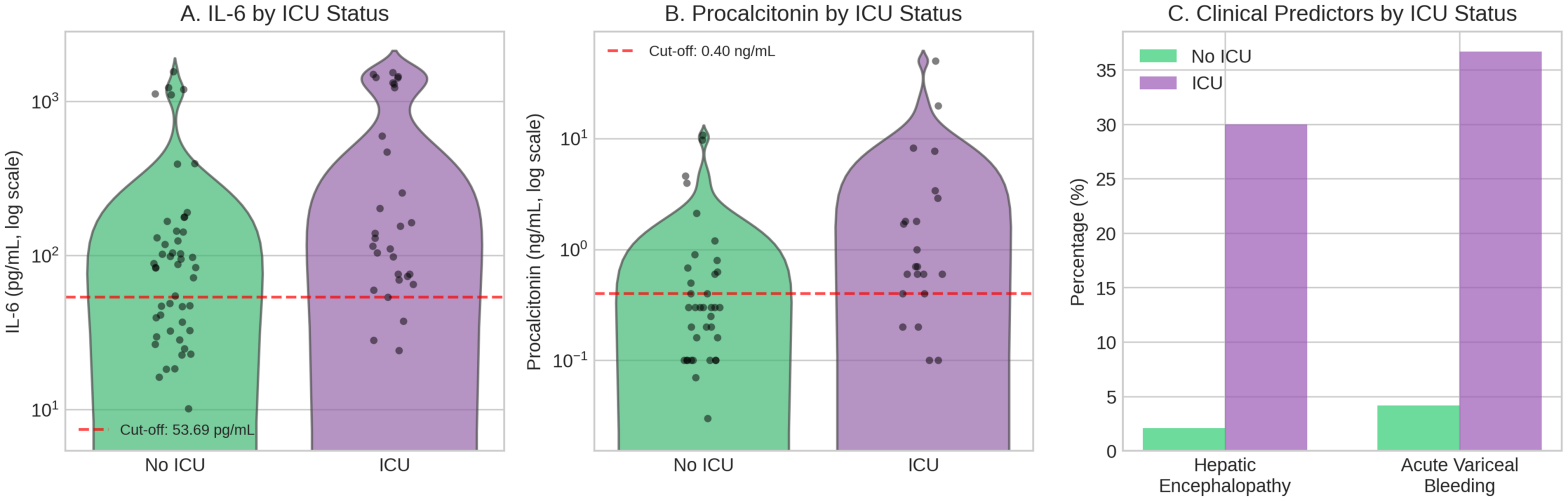
Predictor distributions by ICU admission status. **(A)** Interleukin-6 (IL-6) and **(B)** procalcitonin shown as violin plots with individual data points stratified by ICU admission (*n* = 30) versus no ICU admission (*n* = 48). Procalcitonin data available for 60 patients. Y-axes displayed on logarithmic scale. Dashed red lines indicate ROC-derived cut-off values (IL-6 ≥ 53.69 pg./mL; procalcitonin ≥0.40 ng/mL). **(C)** Prevalence of hepatic encephalopathy and acute variceal bleeding by ICU status, showing markedly higher rates among ICU-admitted patients.

Thirty (38.5%) patients required ICU admission. Comprehensive univariable analysis (Supplementary Table 4) identified eight continuous variables significantly associated with ICU admission: procalcitonin (median 0.70 vs. 0.30 ng/mL, *p* = 0.002), IL-6 (134.58 vs. 86.03 pg./mL, *p* = 0.006), sodium (mean 133.10 vs. 129.88 mEq/L, *p* = 0.026), urea (56.29 vs. 40.03 mg/dL, *p* = 0.040), BUN (26.27 vs. 18.68 mg/dL, *p* = 0.040), INR (2.09 vs. 1.81, *p* = 0.043), MCP-1 (374.51 vs. 279.35 pg./mL, *p* = 0.045), and PT (27.31 vs. 23.88 s, *p* = 0.050), alongside three categorical variables: hepatic encephalopathy (30.0% vs. 2.1%, *p* = 0.001) and acute variceal bleeding (36.7% vs. 4.2%, *p* = 0.001). However, traditional scores like MELD3 (25.40 vs. 24.98, *p* = 0.809) and Child-Pugh (9.17 vs. 8.69, *p* = 0.197) showed no significant association. ROC analysis revealed procalcitonin as the strongest predictor (AUC 0.730, cut-off ≥0.40 ng/mL, sensitivity 81.8%, specificity 63.2%), followed by IL-6 (AUC 0.680, cut-off ≥53.69 pg./mL). Multivariable logistic regression identified four independent predictors of ICU admission: hepatic encephalopathy (adjusted OR 21.14, 95% CI 2.42–184.80, *p* = 0.001), acute variceal bleeding (all patients required ICU, *p* = 0.001), procalcitonin ≥0.40 ng/mL (adjusted OR 7.71, 95% CI 2.17–27.39, *p* = 0.010), and IL-6 ≥ 53.69 pg./mL (adjusted OR 12.31, 95% CI 1.50–101.16, *p* = 0.046). While INR and sodium were significant in univariable analysis (*p* = 0.043 and *p* = 0.026, respectively), they lost significance after multivariable adjustment and were not retained in the final model. Based on these findings, we developed *The Sick Cirrhosis Patient Score Part B for ICU Risk* (Table 2; Figure 2), a point-based system (range 0–9) that stratified patients into low (0–2 points, 11.1% ICU rate), moderate (3–4 points, 38.1% ICU rate), and high-risk (≥5 points, 91.7% ICU rate) categories. The model demonstrated excellent discrimination with a C-statistic of 0.82, sensitivity of 86.4%, specificity of 63.2% and notably high negative predictive value of 88.9% at a cut-off score ≥3, suggesting its utility for identifying patients who can be safely managed outside the ICU setting. For ICU admission, log-transformed IL-6 (OR 1.94, 95% CI: 1.19–3.19, *p* = 0.008) and procalcitonin (OR 2.45, 95% CI: 1.27–4.72, *p* = 0.007) remained significant continuous predictors. Repeated stratified k-fold cross-validation (10 repeats × 5 folds) demonstrated robust and stable performance with a cross-validated AUC of 0.887 (95% CI: 0.715–0.997), essentially equivalent to the apparent AUC of 0.885 (optimism: −0.002). This consistency between apparent and cross-validated performance suggests the ICU prediction model is not overfit to the derivation data.

**Table 2:** The Sick Cirrhosis Patient Score Part B for ICU risk.

Strongest individual predictors
Hepatic encephalopathy: 21-fold increased odds of ICU Acute variceal bleeding: 100% ICU requirement Procalcitonin ≥0.40: 8-fold increased odds, best biomarker IL-6 ≥ 53.69: 12-fold increased odds when elevated

Point assignment	HE: 3, AVB: 3, PCT≥0.40: 2, IL-6 ≥ 53.69: 1		Total: 0–9
Risk categories	Low (0–2): 11–15% ICU rate	Moderate (3–4): 35–45% ICU rate	High (≥ 5): 85–95% ICU rate

Model performance metrics		
Discrimination	C-statistic: 0.82 (95% CI: 0.74–0.90)	Excellent
At cut-off ≥3 points	Sensitivity: 86.4%	High
	Specificity: 63.2%	Moderate
	PPV: 57.6%	Moderate
	NPV: 88.9%	Excellent
	Accuracy: 71.7%	Good
Calibration	Hosmer-Lemeshow χ^2^ = 4.23, *p* = 0.52	Good fit

HE, hepatic encephalopathy; AVB, acute variceal bleeding; PCT, procalcitonin; IL-6, interleukin-6; ICU, intensive care unit; PPV, positive predictive value; NPV, negative predictive value; CI, confidence interval; χ^2^, chi-square (chi-squared statistic).

### 12–24-month mortality predictors

**Figure 3:**
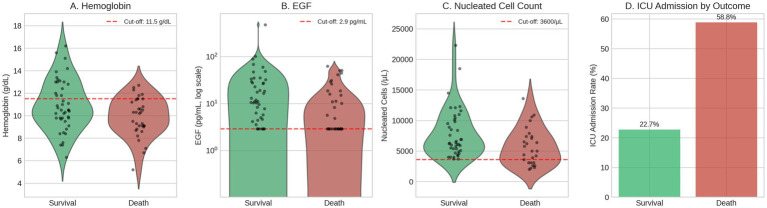
Biomarker distributions by 12–24-month mortality status. Violin plots with individual data points showing **(A)** hemoglobin, **(B)** epidermal growth factor (EGF, logarithmic scale), and **(C)** absolute nucleated cell count stratified by survival (*n* = 44) versus death (*n* = 34). Nucleated cell count available for 69 patients. Dashed red lines indicate ROC-derived cut-off values (hemoglobin ≤11.5 g/dL; EGF ≤ 2.9 pg./mL; nucleated cells ≤3,600/L). **(D)** ICU admission rates by mortality outcome, demonstrating higher ICU utilization among non-survivors.

The overall mortality rate was 43.6% (34/78) (Supplementary Table 5). Multivariate logistic regression analysis of 69 patients with complete data revealed four independent predictors of mortality: hemoglobin ≤11.5 g/dL (OR 7.69, 95% CI: 1.60–36.94, *p* = 0.011), epidermal growth factor ≤2.9 pg./mL (OR 5.57, 95% CI: 1.77–17.55, *p* = 0.003), ICU admission during index hospitalization (OR 5.44, 95% CI: 1.89–15.63, *p* = 0.002), and absolute nucleated cell count ≤3,600/μL (*p* < 0.001, with 100% mortality below this threshold indicating perfect separation). These variables were incorporated into a clinical risk scoring system (*The Sick Cirrhosis Patient Score Part C for Death After Hospitalization*, Table 3; Figure 3) assigning 5 points for nucleated cells ≤3,600/μL, 3 points each for ICU admission and EGF ≤ 2.9 pg./mL, and 2 points for hemoglobin ≤11.5 g/dL. The model demonstrated excellent discrimination with a C-statistic of 0.821, effectively stratifying patients into low (0–2 points, 8.3% mortality), moderate (3–5 points, 20.7% mortality), high (6–8 points, 57.9% mortality), and very high risk (≥9 points, 100% mortality) categories. Notably, the combination of ICU admission and immune dysfunction markers (low nucleated cells and EGF) emerged as particularly powerful predictors, suggesting that acute disease severity and impaired regenerative/immune capacity are critical determinants of long-term survival in hospitalized cirrhosis patients. For mortality, lower hemoglobin (OR 0.58 per SD increase, 95% CI: 0.35–0.95, *p* = 0.031), lower EGF (OR 0.61, 95% CI: 0.38–0.99, *p* = 0.047), and lower nucleated cell count (OR 0.49, 95% CI: 0.25–0.93, *p* = 0.030) were significantly associated with death. Notably, traditional scores (MELD, CTP) showed no significant continuous association with mortality in univariable analysis (*p* > 0.29), consistent with their poor discrimination observed in ROC analysis. Cross-validation using 10 repeats of 5-fold stratified sampling yielded a mean AUC of 0.815 (95% CI: 0.568–0.977), identical to the apparent AUC of 0.815 with no detectable optimism (Supplementary Table 6). The wide confidence interval appropriately reflects uncertainty inherent to the sample size (*n* = 69, 27 events) rather than model instability.

**Table 3:** The Sick Cirrhosis Patient Score Part C for death after hospitalization and action plan.

Point allocation		
Risk factor	Threshold	Points
ICU admission	Yes	3
Absolute nucleated cells	≤3,600/μL	5
EGF	≤2.9 pg./mL	3
Hemoglobin	≤11.5 g/dL	2
Maximum possible score		13

Risk stratification and observed outcomes					
Risk category	Score range	*N* (%)	Deaths	Observed mortality	Predicted mortality
Low	0–2	12 (17.4)	1	8.3%	8–10%
Moderate	3–5	29 (42.0)	6	20.7%	20–25%
High	6–8	19 (27.5)	11	57.9%	60–70%
Very high	≥9	9 (13.0)	9	100.0%	>85%

ICU, intensive care unit; EGF, epidermal growth factor.

### Comparison with traditional severity scores

Traditional severity scores demonstrated limited discrimination for the study outcomes. For 12–24-month mortality, MELD3 achieved an AUC of 0.534 (95% CI: 0.404–0.658) and CTP achieved 0.553 (95% CI: 0.427–0.671), compared to 0.815 for the Sick Cirrhosis Patient Score Part C. For infection at hospitalization, MELD3 AUC was 0.650 (95% CI: 0.500–0.782) and CTP was 0.629 (95% CI: 0.474–0.768), versus 0.759 for Part A. For ICU admission, MELD3 showed near-chance discrimination (AUC 0.510, 95% CI: 0.375–0.640) with CTP performing marginally better (0.581, 95% CI: 0.451–0.721), compared to 0.885 for Part B. These findings underscore the limited utility of traditional scores, which primarily reflect hepatic synthetic function and renal dysfunction, for predicting inflammatory and immune-mediated complications in hospitalized cirrhosis patients. The incremental discrimination provided by the biomarker-based scores ranged from +0.109 to +0.375 AUC units compared to MELD3 (Supplementary Table 7).

## Discussion

**Figure 4:**
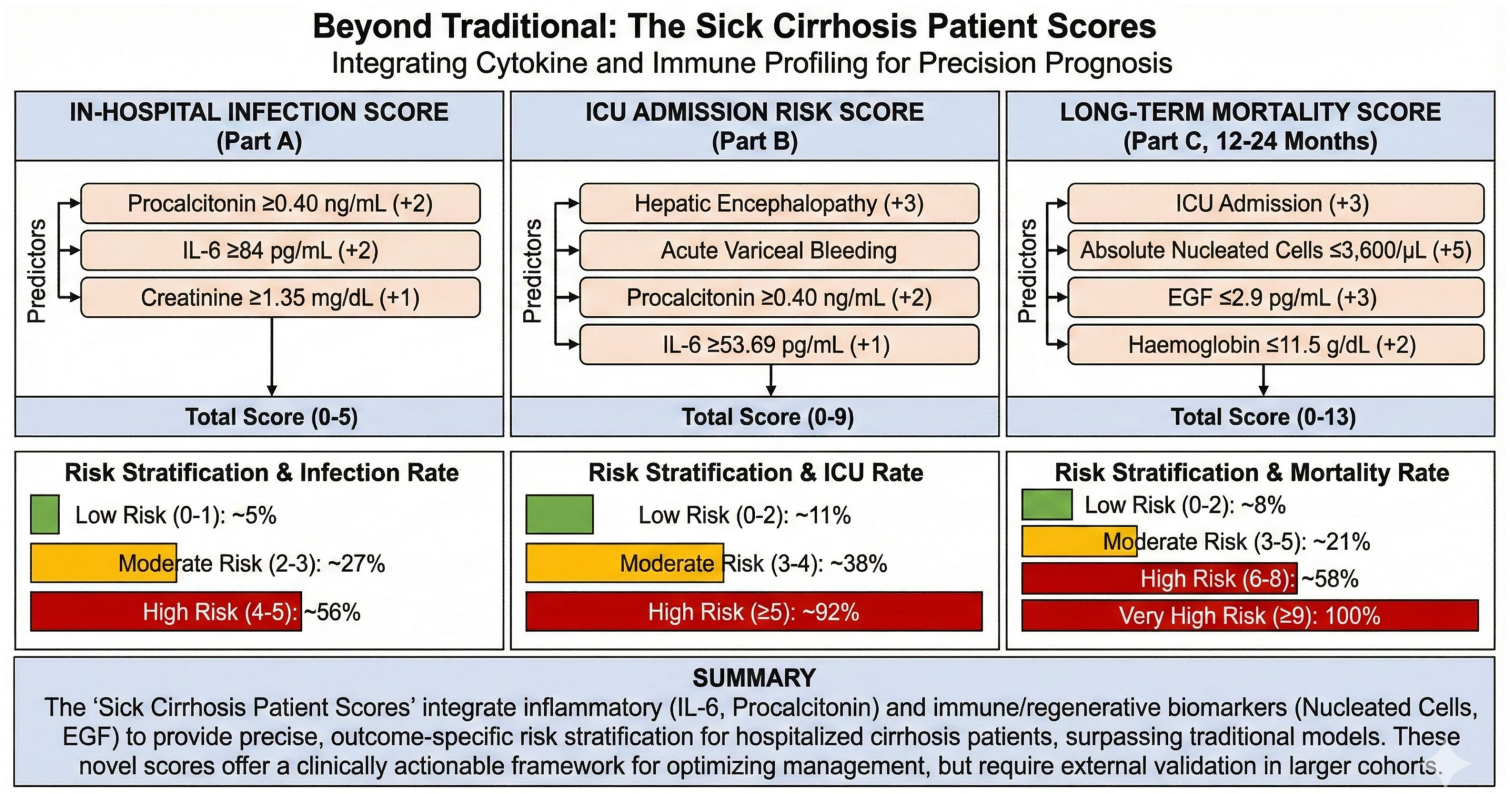
Study summary – independent predictors of infection at admission, need for ICU admission, and death after hospitalization in the long term, along with an algorithmic approach to hospitalized cirrhosis patients, which remains to be validated in prospective large cohorts.

This study demonstrated that a comprehensive assessment of inflammatory and immune biomarkers in hospitalized patients with cirrhosis could significantly refine risk stratification for distinct clinical outcomes: infection, ICU admission, and long-term mortality. Our central contribution was the development of three novel, outcome-specific scoring systems – The ‘Sick Cirrhosis Patient Scores’ – that integrated these biomarkers to provide clinically actionable prognostic information beyond that offered by traditional scores like the MELD and CTP (Figure 4). The limitations of MELD and CTP scores were well-recognized; they primarily reflected the severity of hepatic and renal dysfunction but failed to capture the dynamic state of systemic inflammation that was a key driver of acute decompensation and mortality (15–17). This fueled a search for more dynamic markers and other inflammation-based scores, to better capture this crucial pathophysiological dimension (12).

Our work advanced this paradigm by moving beyond a “one-size-fits-all” prognostic model. By developing separate scores for infection, ICU admission, and mortality, we acknowledged that the underlying pathophysiology and temporal urgency of these endpoints were different. The study’s results showed that the strongest predictors for short-term events like infection and ICU admission were acute inflammatory markers, including interleukin-6 (IL-6) and procalcitonin, which aligned with the pathophysiology of an acute inflammatory “storm” triggered by an insult. Conversely, the most potent predictors for long-term (between 12–24 month) mortality were markers reflecting a state of systemic host failure: immune paresis, indicated by a low absolute nucleated cell count, and an exhausted regenerative capacity, suggested by low epidermal growth factor EGF. This divergence implied that surviving the initial inflammatory insult was a necessary but not sufficient condition for long-term survival; the patient’s underlying immune and regenerative reserve ultimately dictated their long-term trajectory. Therefore, a single score attempting to capture all these dimensions would likely have been less precise than a modular system that allowed clinicians to ask specific questions, such as, “What is my patient’s risk of infection today?” versus “What is my patient’s likelihood of survival at one year?” This tailored approach represented a tangible step toward precision prognostication in clinical hepatology.

Our multivariable analysis confirmed that both IL-6 and procalcitonin were potent, independent predictors of infection and ICU admission in hospitalized cirrhosis patients. This finding was consistent with a large body of literature establishing these as key biomarkers in this population. IL-6 is a pleiotropic cytokine central to the acute phase response; its levels are known to be elevated in cirrhosis and correlated with disease severity, complications like hepatic encephalopathy, and portal hypertension (18). While IL-6 may be non-specifically elevated in cirrhosis due to baseline sterile inflammation from gut bacterial translocation and hepatic dysfunction, its inclusion in our scoring systems is justified by its central role in acute phase dynamics, its temporal advantage over procalcitonin in early infection detection, and its synergistic value when combined with more specific markers such as procalcitonin and creatinine. Similarly, procalcitonin is a well-validated marker for discriminating bacterial infection from sterile inflammation and predicting adverse outcomes (19). Our finding that procalcitonin independently predicted ICU admission was directly corroborated by studies that had identified it as the sole independent predictor in multivariate models designed to assess this specific outcome.

The diagnostic accuracy of IL-6 for infection in our study, was robust, but notably lower than the pooled AUC of 0.94 reported in a meta-analysis (20). This apparent discrepancy was not a weakness of the marker but rather a reflection of our study’s specific high-risk population. The cohort in our study was comprised of severely ill, hospitalized patients with a mean MELD3 score of 25.14, who exhibited a high degree of baseline “sterile” inflammation possibly due to factors like gut bacterial translocation and the release of damage-associated molecular patterns. This state, described in other studies as a combination of “immune paresis and excessive inflammatory response,” resulted in chronically elevated IL-6 levels even without an overt bacterial infection (21). The meta-analysis likely included a broader spectrum of patients, where the contrast between infected individuals and uninfected controls would have been much starker, leading to a higher calculated AUC. In our cohort, the diagnostic challenge was not distinguishing infection from health, but distinguishing a new infection from severe, pre-existing systemic inflammation. In this difficult clinical context, an AUC of 0.707 was clinically meaningful.

The true clinical value of our scores incorporating these markers were in their high negative predictive value NPV and the potential temporal advantage offered by IL-6. Studies had demonstrated that IL-6 levels could peak at the onset of fever, often 24 h before procalcitonin, making it an invaluable early diagnostic marker (22). Our ‘Sick Cirrhosis Patient Score Part A for Infection’ achieved an NPV of 90.0%. This suggested that a low score could have provided clinicians with a high degree of confidence to defer or de-escalate empiric antibiotics, a critical consideration in the era of antimicrobial resistance. Similarly, the 88.9% NPV of the ‘Sick Cirrhosis Patient Score Part B for ICU Risk’ could help rationalize resource allocation by identifying patients who could be safely managed on a general medical ward.

The identification of an absolute nucleated cell count ≤3,600/μL as a powerful predictor of 12–24-month mortality was a key and novel finding of this study. The observation of 100% mortality below this threshold (“perfect separation”) in our cohort, while requiring cautious interpretation due to sample size, pointed to a state of profound systemic failure. This finding had to be understood within the dual framework of Cirrhosis-Associated Immune Dysfunction (CAID), which encompassed both hyper-inflammation and immunodeficiency, or immune paresis. While much research had focused on markers of relative inflammation, such as the NLR, our data highlighted the lethal consequences of the immunodeficiency pole of this syndrome (23). Leukopenia was a known adverse prognostic sign in cirrhosis, often associated with hypersplenism but also reflecting deeper systemic pathology. A low absolute nucleated cell count may have represented a more advanced stage of immune collapse. A profoundly low absolute nucleated cell count suggested a global failure of hematopoiesis affecting all cell lines. This was not merely an imbalance but a comprehensive failure of the host’s ability to mount any effective immune response. This state of bone marrow exhaustion or terminal immune paresis rendered the patient exquisitely vulnerable to any subsequent stressor, which explained the observed 100% mortality in this subgroup. It likely represented a pre-terminal state where the capacity for host defence was irreversibly lost.

The identification of low serum EGF as an independent predictor of long-term mortality (OR 5.57) represents a hypothesis-generating finding of this study. The EGF receptor (EGFR) signaling axis is known to mediate hepatocyte proliferation and liver regeneration (24). We speculate that low circulating EGF in patients with severe hepatic decompensation may reflect exhausted regenerative capacity, though our single cross-sectional measurement cannot establish causality or temporal relationships. The apparent paradox between our finding (low EGF predicting mortality from liver failure) and literature associating high EGF/EGFR activity with hepatocellular carcinoma progression likely reflects context-dependent roles of this pathway, but this requires dedicated mechanistic investigation beyond the scope of our prognostic study (25). Future studies with serial EGF measurements and correlation with histological regenerative activity would help clarify this association.

Our study confirmed that anemia was a powerful independent predictor of long-term mortality, with an odds ratio of 7.69. This result was highly consistent with previous research. Anemia was extremely common in patients with cirrhosis and had been repeatedly identified as an independent risk factor for hepatic decompensation and mortality in multiple cohorts (26). A large multicenter study specifically identified severe anemia as an independent predictor of 1-year mortality, with a hazard ratio of 1.610 (27). The odds ratio observed in our study was notably high. This suggested that in the context of our multivariable model, anemia was capturing a dimension of disease severity not fully accounted for by the other variables. Anemia in cirrhosis was multifactorial, resulting from acute or chronic bleeding, hypersplenism, nutritional deficiencies, and inflammation-mediated suppression of erythropoiesis. Our model for mortality already included markers of acute illness severity (ICU admission) and fundamental biological failure (low EGF, low nucleated cells). The fact that anemia remained a highly significant predictor on top of these factors implied it was acting as an integrated proxy for multiple chronic insults – such as malnutrition, chronic inflammation, and bone marrow dysfunction – that cumulatively eroded the patient’s physiological reserve and contributed to their long-term demise.

The development of three distinct, point-based scores was a pragmatic and unique feature of our study. This modular design reflected clinical reality, where prognostication was not a single question but a series of specific queries that evolved over the course of a patient’s hospitalization. The scores were designed for ease of use, translating complex biomarker data into simple, stratified risk categories (low, moderate, high) that could be readily applied at the bedside. The performance and potential impact of these scores were significant. For the ‘Sick Cirrhosis Patient Score Part A for Infection’ and ‘Part B for ICU Risk’, the excellent discrimination was underscored by their high NPVs. In an era of increasing antibiotic resistance and constrained ICU resources, tools that could reliably identify low-risk patients were of immense value. They could support evidence-based decisions to avoid unnecessary antibiotics and ICU admissions, potentially improving patient outcomes and optimizing healthcare utilization. The ‘Sick Cirrhosis Patient Score Part C for Death’ demonstrated excellent discrimination for long-term mortality. Its ability to stratify patients into groups with mortality rates ranging from 8.3 to 100% provided a powerful tool for patient counseling, facilitating crucial discussions about goals of care, the appropriateness of aggressive interventions, and the potential need for expedited liver transplant evaluation. This moved beyond the primary function of the MELD3 score, which was designed for organ allocation rather than nuanced, long-term prognostication.

While serum creatinine is a component of the MELD score, it is important to note that MELD was designed for liver transplant allocation rather than predicting infection or ICU admission in hospitalized patients. In our Sick Cirrhosis Patient Score Part A for Infection, creatinine is used with a different, ROC-optimized threshold (≥1.35 mg/dL) and, critically, is combined with inflammatory biomarkers (IL-6 and procalcitonin) that are not captured by MELD. This integration of end-organ dysfunction with active inflammatory markers represents the conceptual advance over traditional scores. Furthermore, creatinine is included only in the infection score; the ICU and mortality scores employ entirely different predictor combinations, reflecting our outcome-specific modular design.

The wide confidence intervals observed in cross-validation, particularly for the infection score (95% CI: 0.354–1.000), reflect the fundamental statistical challenge of developing prediction models with limited sample sizes and modest event counts. These intervals represent honest uncertainty quantification rather than model failure. Importantly, the mean cross-validated AUCs closely approximated apparent performance across all models (optimism range: −0.002 to +0.009), indicating that while precision is limited, the point estimates are reliable. The variability in individual fold performance is expected when test sets contain only 12–14 patients per fold, of whom only 4–6 experience the outcome. This underscores the critical need for external validation in larger, independent cohorts to obtain more precise performance estimates.

The proposed Sick Cirrhosis Patient Scores should be considered hypothesis-generating tools at this stage of development. Should external validation confirm their discriminative ability, potential future applications might include: stratification of patients for intensity of monitoring, identification of low-risk patients who may be suitable for ward-level care, flagging of high-risk patients for early specialist review, and prognostic counseling to inform goals-of-care discussions. However, any such clinical applications remain speculative pending rigorous validation in independent, multicenter cohorts. We explicitly caution against clinical implementation based solely on this derivation study.

This study had several notable strengths. The use of a comprehensive panel of inflammatory and immune markers, measured simultaneously with high-quality, standardized laboratory techniques, provided a uniquely detailed snapshot of the patient’s biological state at a single point in time. The development of simple, clinically intuitive scoring systems and the identification of novel, biologically plausible predictors like EGF and absolute nucleated cell count were significant contributions to the field.

We had to, however, acknowledge several important limitations. First, the study was a retrospective analysis, conducted at a single center with a relatively small cohort of 78 patients. This limited the generalizability of our findings and increased the risk of statistical overfitting, where the models might perform less well in other populations. The wide confidence intervals for some predictors in the multivariable models and the “perfect separation” observed for certain significant predictors underscored the need for caution in interpreting the precise magnitude of these effects. Second, our use of complete case analysis for multivariable modeling, which reduced the sample size to between 60 and 69 patients for different models, may have introduced selection bias. Finally, and most critically, these novel scoring systems were developed and tested in the same dataset. They require rigorous external validation in larger, independent, multicenter cohorts before they could be recommended for widespread clinical use. Several additional limitations also warrant discussion. The use of logistic regression rather than time-to-event analysis for the mortality outcome is a methodological limitation; our dataset lacked exact dates of death required for Cox regression or Kaplan–Meier analysis. Future prospective studies should capture precise follow-up times to enable proper survival analysis. The dichotomization of continuous biomarkers using ROC-derived cut-offs from the derivation dataset likely inflates apparent performance and discards potentially useful continuous information; these thresholds should be considered exploratory rather than validated clinical cut-points. The absence of a healthy control group or compensated cirrhosis comparator limits our ability to contextualize whether the observed biomarker profiles are specific to decompensated disease. Sample collection timing relative to interventions (antibiotics, transfusion, paracentesis) was not fully standardized, which may introduce variability in biomarker measurements. The absence of liver transplantation in our cohort, while eliminating transplant-related censoring, may introduce survival bias toward transplant-ineligible patients and limits generalizability to centers with active transplant programs.

In conclusion, this study provided initial evidence that the integration of biomarkers reflecting systemic inflammation (IL-6, procalcitonin), immune paresis (absolute nucleated cell count), and regenerative capacity (EGF) could substantially enhance risk stratification for hospitalized patients with cirrhosis. The proposed ‘Sick Cirrhosis Patient Scores’ offered a novel, outcome-specific framework that moved beyond traditional static measures of organ dysfunction. The findings from this study laid the groundwork for several critical avenues of future research. The immediate and most crucial next step is the external validation of the ‘Sick Cirrhosis Patient Scores’ in large, prospective, cohorts to establish their generalizability and refine their calibration. Randomized controlled trials could investigate whether modulating hyper-inflammation with anti-cytokine therapies in carefully selected patients, or promoting liver regeneration by targeting the EGF pathway, can improve clinical outcomes. Finally, future studies should incorporate serial measurements of these biomarkers to map their temporal trajectories during hospitalization. Understanding these dynamic changes could allow for real-time monitoring of treatment response and the early identification of patients who were failing to improve, enabling more timely and personalized interventions.

## Data Availability

The original contributions presented in the study are included in the article/Supplementary material, further inquiries can be directed to the corresponding author.
