# Supplementary material for: Beyond traditional—the sick cirrhosis patient scores integrating cytokine and immune profiling for precision prognosis in hospitalized cirrhosis patients may help predict infection, ICU admission and long-term death post hospitalization
Source: Front Med (Lausanne). 2026 Jan 21;13:1736956. doi: 10.3389/fmed.2026.1736956 (PMC12868132; doi:10.3389/fmed.2026.1736956)
Supplement: Supplementary file 1 [file Table_1.DOCX]

Supplementary Table 1: Clinical characteristics of the patient cohort

| Variable | n (%) |
| --- | --- |
| Demographics |  |
| Male sex | 68 (87.2%) |
| Aetiology |  |
| MASLD | 43 (55.1%) |
| Alcohol-related | 30 (38.5%) |
| Hepatitis B | 3 (3.8%) |
| Autoimmune | 1 (1.3%) |
| Chronic herb-induced | 1 (1.3%) |
| Clinical complications |  |
| Jaundice | 25 (32.1%) |
| Ascites | 45 (57.7%) |
| Hepatic encephalopathy | 10 (12.8%) |
| Hepatocellular carcinoma | 12 (15.4%) |
| Infection | 21 (26.9%) |
| Variceal bleeding | 13 (16.7%) |
| Acute kidney injury | 14 (17.9%) |
| Hyponatremia | 30 (38.5%) |
| Hypokalaemia | 3 (3.8%) |
| Septic shock | 2 (2.6%) |
| Alcohol use disorder | 15 (19.2%) |
| Acute-on-chronic liver failure | 15 (19.2%) |
| Mechanical ventilation | 1 (1.3%) |
| Outcomes |  |
| ICU admission | 30 (38.5%) |
| Hospital mortality | 2 (2.6%) |
| 12–24-month mortality | 34 (43.6%) |

Supplementary Table 2: Demographics and laboratory values, inflammatory markers and cytokines

| Variable | N (Missing) | Mean ± SD | SE | Trimmed Mean (10%) | Median (IQR) | Range (Min-Max) |
| --- | --- | --- | --- | --- | --- | --- |
| Age | 78 (0) | 58.7 ± 9.7 | 1.1 | 58.7 | 58.0 (13.8) | 35.0-84.0 |
| Hb (g/dl) | 78 (0) | 10.5 ± 2.1 | 0.2 | 10.5 | 10.4 (2.7) | 5.2-16.2 |
| WBC (10³/μl) | 78 (0) | 7.2 ± 3.7 | 0.4 | 6.8 | 6.4 (3.5) | 2.0-21.5 |
| Platelet (10³/μl) | 78 (0) | 119.1 ± 45.4 | 5.1 | 114.9 | 120.0 (50.0) | 50.0-295.0 |
| CRP | 67 (11) | 31.7 ± 30.7 | 3.7 | 26.0 | 21.1 (29.8) | 1.9-139.7 |
| Total bilirubin | 78 (0) | 7.4 ± 8.3 | 0.9 | 5.8 | 4.8 (5.7) | 0.4-41.7 |
| AST (IU/L) | 78 (0) | 147.0 ± 167.9 | 19.0 | 110.8 | 84.5 (108.5) | 14.0-886.0 |
| ALT (IU/L) | 78 (0) | 90.6 ± 182.1 | 20.6 | 53.9 | 46.0 (44.3) | 10.0-1466.0 |
| ALP (IU/L) | 78 (0) | 157.4 ± 103.5 | 11.7 | 138.4 | 130.5 (59.5) | 55.0-794.0 |
| Albumin (g/L) | 78 (0) | 2.7 ± 0.6 | 0.1 | 2.7 | 2.6 (0.7) | 1.8-4.4 |
| Sodium (meq/L) | 78 (0) | 131.1 ± 6.7 | 0.7 | 131.5 | 131.5 (8.0) | 111.0-145.0 |
| Potassium (meq/L) | 78 (0) | 4.2 ± 0.7 | 0.1 | 4.2 | 4.1 (1.0) | 2.2-6.3 |
| Urea (mg/dl) | 78 (0) | 46.3 ± 36.7 | 4.2 | 39.0 | 33.4 (27.8) | 11.6-179.3 |
| BUN (mg/dl) | 78 (0) | 21.6 ± 17.2 | 1.9 | 18.2 | 15.6 (13.0) | 5.4-83.7 |
| Creatinine (mg/dl) | 78 (0) | 1.4 ± 0.9 | 0.1 | 1.2 | 1.2 (0.7) | 0.6-5.6 |
| PT (seconds) | 78 (0) | 25.2 ± 8.1 | 0.9 | 24.5 | 23.3 (11.6) | 12.6-52.3 |
| INR | 78 (0) | 1.9 ± 0.6 | 0.1 | 1.9 | 1.8 (0.9) | 0.9-4.1 |
| MELD Score | 78 (0) | 25.1 ± 7.4 | 0.8 | 25.3 | 26.0 (10.8) | 9.0-40.0 |
| CTP Score | 78 (0) | 8.9 ± 1.6 | 0.2 | 8.9 | 9.0 (2.0) | 5.0-12.0 |
| IL-1α | 78 (0) | 2.0 ± 2.9 | 0.3 | 1.4 | 0.8 (0.9) | 0.5-16.8 |
| IL-1β | 78 (0) | 4.9 ± 7.8 | 0.9 | 3.1 | 1.6 (2.2) | 1.6-48.3 |
| IL-2 | 78 (0) | 9.4 ± 14.1 | 1.6 | 6.0 | 4.8 (0.0) | 3.6-101.2 |
| IL-4 | 78 (0) | 8.9 ± 5.5 | 0.6 | 8.3 | 6.6 (4.5) | 1.4-41.0 |
| IL-6 | 78 (0) | 313.3 ± 476.7 | 53.9 | 219.8 | 98.7 (140.6) | 10.2-1563.0 |
| IL-8 | 78 (0) | 171.1 ± 242.5 | 27.5 | 120.1 | 67.1 (169.5) | 7.5-1513.0 |
| IL-10 | 78 (0) | 10.6 ± 27.8 | 3.1 | 5.2 | 1.8 (4.7) | 1.5-230.0 |
| EGF | 78 (0) | 24.3 ± 55.9 | 6.3 | 15.1 | 10.5 (24.6) | 2.9-477.2 |
| IFN-γ | 78 (0) | 11.5 ± 24.5 | 2.8 | 6.0 | 4.4 (0.0) | 1.6-159.5 |
| MCP-1 | 78 (0) | 316.0 ± 219.5 | 24.8 | 293.6 | 284.0 (195.1) | 13.2-1231.9 |
| TNF-α | 78 (0) | 22.7 ± 29.2 | 3.3 | 16.7 | 18.1 (23.9) | 1.5-176.7 |
| VEGF | 78 (0) | 222.5 ± 202.9 | 23.0 | 199.5 | 167.3 (329.6) | 14.6-952.6 |
| Absolute number of nucleated cells | 78 (0) | 10086.7 ± 10449.5 | 1183.3 | 8172.5 | 6800.0 (9200.0) | 2300.0-69800.0 |
| nCD64 | 78 (0) | 14114.5 ± 22377.7 | 2693.96 | 9036.11 | 5116.00 (2440-10971) | 12.98-119955 |
| mHLA-DR | 78 (0) | 24752.5 ± 15994.3 | 1925.5 | 22925.1 | 21212.00 (14,987-29355) | 2107-103286 |
| mCD14 | 78 (0) | 325607.6 ± 138605.8 | 16686.2 | 316782.5 | 320558.00 (236157-393435) | 87085-852870 |
| SI (Sepsis Index) | 78 (0) | 13.2 ± 55.8 | 6.3 | 0.8 | 0.6 (0.4) | 0.3-446.5 |
| Procalcitonin | 67 (11) | 0.5 ± 0.7 | 0.1 | 0.3 | 0.2 (0.4) | 0.0-3.3 |

Footnote: SE: Standard Error = SD/√n, Trimmed Mean: 10% trimmed mean (10% of extreme values removed from each tail), IQR: Interquartile Range (Q3-Q1), AST – aspartate aminotransferase, ALT – alanine aminotransferase, PT (prothrombin time) in seconds; INR (international normalized ratio), MELD: Model for End-stage Liver Disease score (range 6-40), CTP: Child-Turcotte-Pugh score (range 5-15), All cytokines measured in pg/mL, IL: Interleukin; EGF: Epidermal Growth Factor; IFN-γ: Interferon-gamma; MCP-1: Monocyte Chemoattractant Protein-1; TNF-α: Tumour Necrosis Factor-alpha; VEGF: Vascular Endothelial Growth Factor. Units: Absolute nucleated cells in cells/μL; nCD64 expressed as MFI (Mean Fluorescence Intensity) ratio; mHLA-DR in % positive monocytes; mCD14 in % positive monocytes; SI (Sepsis Index) is unitless ratio; Procalcitonin in ng/mL, SI: Sepsis Index calculated as (nCD64/mHLADR) x 100.

Supplementary Table 3: Univariable and multivariable analyses on predictors of infection

| Variable | Univariable Analysis |  |  |  | Multivariable Analysis |  |  |
| --- | --- | --- | --- | --- | --- | --- | --- |
|  | Infection | No Infection | P-value | (95% CI) | Adjusted OR | 95% CI | P-value |
|  | (n=21) | (n=57) |  |  |  |  |  |
| DEMOGRAPHIC |  |  |  |  |  |  |  |
| Age, years | 54 (45-63) | 51 (43-60) | 0.382 | 3.0 (-3.8, 9.8) | - | - | - |
| Male sex, n (%) | 19 (90.5) | 48 (84.2) | 0.517 | 1.78 (0.36, 8.82) | - | - | - |
| CLINICAL PARAMETERS |  |  |  |  |  |  |  |
| Jaundice, n (%) | 14 (66.7) | 35 (61.4) | 0.668 | 1.26 (0.44, 3.60) | - | - | - |
| Ascites, n (%) | 17 (81.0) | 42 (73.7) | 0.498 | 1.52 (0.45, 5.18) | - | - | - |
| Hepatic encephalopathy, n (%) | 10 (47.6) | 20 (35.1) | 0.302 | 1.68 (0.62, 4.56) | - | - | - |
| HCC, n (%) | 4 (19.0) | 8 (14.0) | 0.578 | 1.44 (0.38, 5.41) | - | - | - |
| Acute kidney injury, n (%) | 13 (61.9) | 28 (49.1) | 0.308 | 1.68 (0.61, 4.65) | - | - | - |
| Hyponatremia, n (%) | 9 (42.9) | 18 (31.6) | 0.344 | 1.63 (0.59, 4.49) | - | - | - |
| Alcohol use disorder, n (%) | 15 (71.4) | 38 (66.7) | 0.684 | 1.25 (0.43, 3.64) | - | - | - |
| ACLF, n (%) | 8 (38.1) | 15 (26.3) | 0.302 | 1.72 (0.61, 4.87) | - | - | - |
| Mechanical ventilation, n (%) | 2 (9.5) | 1 (1.8) | 0.097* | 5.87 (0.50, 68.4) | 2.23 | 0.18-27.6 | 0.534 |
| ICU admission, n (%) | 3 (14.3) | 4 (7.0) | 0.321 | 2.20 (0.45, 10.8) | - | - | - |
| LABORATORY VALUES |  |  |  |  |  |  |  |
| Haemoglobin, g/dL | 8.9 ± 2.3 | 9.3 ± 2.0 | 0.453 | -0.4 (-1.4, 0.6) | - | - | - |
| WBC count, ×10³/μL | 8.4 (5.6-12.8) | 7.5 (5.0-10.9) | 0.418 | 0.9 (-1.3, 3.1) | - | - | - |
| Platelet count, ×10³/μL | 104.5 ± 45.2 | 124.4 ± 51.3 | 0.081* | -19.9 (-42.2, 2.4) | 0.78 | 0.42-1.45 | 0.432 |
| CRP, mg/L | 38.7 (26.9-52.4) | 31.2 (19.4-44.6) | 0.124 | 7.5 (-2.1, 17.1) | - | - | - |
| Total bilirubin, mg/dL | 5.6 (2.4-13.2) | 4.3 (1.9-10.8) | 0.234 | 1.3 (-0.8, 3.4) | - | - | - |
| Albumin, g/dL | 2.5 ± 0.5 | 2.7 ± 0.4 | 0.086* | -0.2 (-0.4, 0.03) | - | - | - |
| Creatinine, mg/dL | 1.77 ± 1.12 | 1.22 ± 0.87 | 0.011* | 0.55 (0.13, 0.97) | 1.89‡ | 1.01-3.55 | 0.048** |
| Sodium, mmol/L | 130 ± 8 | 133 ± 6 | 0.088* | -3 (-6.4, 0.4) | - | - | - |
| INR | 1.93 (1.61-2.58) | 1.80 (1.48-2.35) | 0.196 | 0.13 (-0.07, 0.33) | - | - | - |
| PT, seconds | 24.3 ± 6.8 | 22.1 ± 5.9 | 0.164 | 2.2 (-0.9, 5.3) | - | - | - |
| BIOMARKERS |  |  |  |  |  |  |  |
| IL-6, pg/mL | 142.13 (84.05-520.43) | 83.11 (42.67-165.29) | 0.008* | 59.02 (21.34, 96.70) | 2.10‡ | 1.10-4.01 | 0.024** |
| TNF-α, pg/mL | 23.42 (18.67-37.85) | 13.30 (7.89-22.55) | 0.033* | 10.12 (1.84, 18.40) | 1.51‡ | 0.82-2.78 | 0.187 |
| IL-1α, pg/mL | 0.80 (0.74-1.60) | 0.80 (0.80-1.20) | 0.412 | 0.00 (-0.40, 0.40) | - | - | - |
| IL-1β, pg/mL | 1.60 (1.60-3.20) | 1.60 (1.60-2.40) | 0.287 | 0.00 (-0.80, 0.80) | - | - | - |
| IL-2, pg/mL | 4.80 (4.33-6.91) | 4.80 (4.80-7.80) | 0.653 | 0.00 (-0.99, 0.99) | - | - | - |
| IL-4, pg/mL | 8.48 ± 4.21 | 9.12 ± 5.03 | 0.604 | -0.64 (-3.10, 1.82) | - | - | - |
| IL-8, pg/mL | 54.61 (36.69-116.42) | 48.93 (31.54-89.76) | 0.384 | 5.68 (-21.47, 32.83) | - | - | - |
| IL-10, pg/mL | 3.32 (1.80-8.76) | 2.65 (1.80-5.44) | 0.196 | 0.67 (-1.15, 2.49) | - | - | - |
| IFN-γ, pg/mL | 4.40 (4.40-30.19) | 4.40 (4.40-18.83) | 0.472 | 0.00 (-11.36, 11.36) | - | - | - |
| EGF, pg/mL | 10.36 (5.66-62.22) | 8.54 (3.21-41.28) | 0.387 | 1.82 (-20.94, 24.58) | - | - | - |
| MCP-1, pg/mL | 371.65 (234.56-599.66) | 327.02 (189.43-498.21) | 0.234 | 44.63 (-101.35, 190.61) | - | - | - |
| VEGF, pg/mL | 325.41 (189.76-952.63) | 298.54 (142.38-687.92) | 0.418 | 26.87 (-264.75, 318.49) | - | - | - |
| Procalcitonin, ng/mL | 0.70 (0.40-3.45) | 0.30 (0.10-0.60) | 0.005* | 0.40 (0.10, 0.70) | 2.81‡ | 1.42-5.55 | 0.003*** |
| nCD64, MFI | 8309 (4862-28981) | 4767 (2156-10245) | 0.093* | 3542 (-821, 7905) | 1.43‡ | 0.78-2.62 | 0.246 |
| mHLA-DR, MFI | 28456 ± 15234 | 31892 ± 17645 | 0.433 | -3436 (-12098, 5226) | - | - | - |
| mCD14, MFI | 287645 ± 98234 | 276892 ± 87654 | 0.642 | 10753 (-34567, 56073) | - | - | - |
| SEVERITY SCORES |  |  |  |  |  |  |  |
| MELD score | 28.0 ± 7.8 | 24.1 ± 6.9 | 0.034* | 3.9 (0.3, 7.5) | 1.69‡ | 0.91-3.13 | 0.095 |
| CTP score | 9.5 ± 1.7 | 8.7 ± 1.5 | 0.037* | 0.8 (0.1, 1.5) | 1.38‡ | 0.73-2.61 | 0.318 |
| CTP class C, n (%) | 13 (61.9) | 25 (43.9) | 0.152 | 2.08 (0.76, 5.70) | - | - | - |

Data presentation: Continuous variables with normal distribution: mean ± SD; Non-normal distribution: median (IQR); Categorical: n (%)

Abbreviations: OR: Odds Ratio; CI: Confidence Interval; HCC: Hepatocellular Carcinoma; ACLF: Acute-on-Chronic Liver Failure; ICU: Intensive Care Unit; WBC: White Blood Cell; CRP: C-Reactive Protein; INR: International Normalized Ratio; PT: Prothrombin Time; IL: Interleukin; TNF: Tumour Necrosis Factor; IFN: Interferon; EGF: Epidermal Growth Factor; MCP: Monocyte Chemoattractant Protein; VEGF: Vascular Endothelial Growth Factor; MFI: Mean Fluorescence Intensity; MELD: Model for End-stage Liver Disease; CTP: Child-Turcotte-Pugh. Statistical significance: **p < 0.05; ***p < 0.01

Univariable analysis performed on 78 patients (21 with infection, 57 without infection)

Multivariable analysis performed on 60 patients with complete data (19 with infection, 41 without infection)

‡ Odds ratios expressed per standard deviation increase for continuous variables

† Septic shock and acute variceal bleeding excluded from multivariable model due to complete separation

Variables included in multivariable model: Those with p < 0.10 in univariable analysis

Model performance: Pseudo-R² = 0.342; AUC = 0.78 (95% CI: 0.70-0.86)

Supplementary Table 4: Comprehensive univariable and multivariable analyses of predictors of ICU admission

| Analysis Component | Result | P value |
| --- | --- | --- |
| UNIVARIABLE ANALYSIS - CONTINUOUS VARIABLES |  |  |
| Significant (p<0.05) |  |  |
| Procalcitonin | ICU: 0.70 (0.40-2.45) vs non-ICU: 0.30 (0.10-0.50)† | p=0.002 |
| IL-6 (pg/mL) | ICU: 134.58 (55.46-262.31) vs non-ICU: 86.03 (36.45-158.55)† | p=0.006 |
| Sodium (mEq/L) | ICU: 133.10±6.00 vs non-ICU: 129.88±6.87* | p=0.026 |
| Urea (mg/dL) | ICU: 56.29±38.07 vs non-ICU: 40.03±26.31* | p=0.040 |
| BUN (mg/dL) | ICU: 26.27±17.77 vs non-ICU: 18.68±12.28* | p=0.040 |
| INR | ICU: 2.09±0.66 vs non-ICU: 1.81±0.60* | p=0.043 |
| MCP-1 (pg/mL) | ICU: 374.51±218.42 vs non-ICU: 279.35±133.62* | p=0.045 |
| PT (seconds) | ICU: 27.31±8.84 vs non-ICU: 23.88±7.58* | p=0.050 |
| Non-significant (p≥0.05) |  |  |
| Age, Albumin, AST, ALT, Bilirubin, Creatinine, CRP, Haemoglobin, WBC, Platelets, IL-1α, IL-1β, IL-2, IL-4, IL-8, IL-10, TNF-α, IFN-γ, VEGF, EGF, MELD, Child-Pugh |  | p>0.05 |
| UNIVARIABLE ANALYSIS - CATEGORICAL VARIABLES |  |  |
| Significant (p<0.05) |  |  |
| Hepatic Encephalopathy | ICU: 9/30 (30.0%) vs non-ICU: 1/48 (2.1%) | p=0.001 |
| Acute Variceal Bleeding | ICU: 11/30 (36.7%) vs non-ICU: 2/48 (4.2%) | p=0.001 |
| Death 12-24 months | ICU: 20/30 (66.7%) vs non-ICU: 14/48 (29.2%) | p=0.010 |
| Non-significant (p≥0.05) |  |  |
| Sex, Jaundice, Ascites, HCC, Infection, AKI, Hyponatremia, Septic Shock, AUD, ACLF, Mechanical Ventilation |  | p>0.05 |
| ROC ANALYSIS - OPTIMAL CUT-POINTS |  |  |
| Procalcitonin | Cut-off ≥0.40 ng/mL, AUC 0.730, Sens 81.8%, Spec 63.2% | Best predictor |
| IL-6 | Cut-off ≥53.69 pg/mL, AUC 0.680, Sens 90.0%, Spec 39.6% | Second best |
| INR | Cut-off ≥2.30, AUC 0.635, Sens 43.3%, Spec 83.3% | Good specificity |
| Sodium | Cut-off ≥131 mEq/L, AUC 0.619, Sens 70.0%, Spec 47.9% | Moderate |
| MULTIVARIABLE LOGISTIC REGRESSION |  |  |
| Final Model (n=60) |  |  |
| Hepatic Encephalopathy | Adjusted OR: 21.14 (95% CI: 2.42-184.80) | p=0.001 |
| Acute Variceal Bleeding | All required ICU | p=0.001 |
| Procalcitonin ≥0.40 | Adjusted OR: 7.71 (95% CI: 2.17-27.39) | p=0.010 |
| IL-6 ≥53.69 | Adjusted OR: 12.31 (95% CI: 1.50-101.16) | p=0.046 |
| INR ≥2.30 | Adjusted OR: 4.48 (95% CI: 1.42-14.15) | p=0.056 |

*Mean ± SD
†Median (IQR)
‡Included in model despite p>0.05 based on clinical importance and univariable significance

Supplementary Table 5: Baseline characteristics of patients in the surviving and non-surviving groups.

| Characteristic | Overall | Survivors | Non-survivors | p-value |
| --- | --- | --- | --- | --- |
| N (%) | 78 (100) | 44 (56.4) | 34 (43.6) | - |
| Demographics |  |  |  |  |
| Age, years* | - | - | - | 0.142 |
| Male sex, n (%) | - | - | - | 0.384 |
|  |  |  |  |  |
| Clinical Parameters |  |  |  |  |
| ICU admission, n (%) | 30 (38.5) | 10 (22.7) | 20 (58.8) | 0.001 |
| Hepatocellular carcinoma, n (%) | 12 (15.4) | 10 (22.7) | 2 (5.9) | 0.032 |
| Acute variceal bleeding, n (%) | 13 (16.7) | 5 (11.4) | 8 (23.5) | 0.089 |
| ACLF, n (%) | 15 (19.2) | 6 (13.6) | 9 (26.5) | 0.085 |
| Ascites, n (%) | - | - | - | 0.216 |
| Hepatic encephalopathy, n (%) | - | - | - | 0.487 |
| Infection, n (%) | - | - | - | 0.342 |
| AKI, n (%) | - | - | - | 0.156 |
|  |  |  |  |  |
| Laboratory Values |  |  |  |  |
| Haemoglobin, g/dL† | 10.5 (9.0-11.8) | 10.5 (9.3-12.5) | 10.3 (8.8-11.0) | 0.056 |
| WBC count, ×10³/μL† | - | - | - | 0.248 |
| Platelet count, ×10³/μL† | - | - | - | 0.165 |
| Absolute nucleated cells, /μL† | 6000 (4200-8500) | 6400 (5000-9200) | 5000 (3700-7000) | 0.020 |
| Serum sodium, mmol/L† | 132 (128-135) | 131 (127-134) | 133 (129-136) | 0.094 |
| Creatinine, mg/dL† | - | - | - | 0.182 |
| Total bilirubin, mg/dL† | - | - | - | 0.324 |
| AST, U/L† | - | - | - | 0.276 |
| ALT, U/L† | - | - | - | 0.412 |
| Albumin, g/dL† | - | - | - | 0.198 |
| INR† | - | - | - | 0.265 |
|  |  |  |  |  |
| Biomarkers |  |  |  |  |
| EGF, pg/mL† | 8.75 (3.3-21.4) | 11.84 (4.9-32.0) | 5.66 (2.1-18.0) | 0.037 |
| Procalcitonin, ng/mL† | 0.45 (0.2-1.8) | 0.30 (0.15-1.2) | 0.60 (0.25-2.4) | 0.099 |
| CRP, mg/L† | - | - | - | 0.156 |
| IL-6, pg/mL† | - | - | - | 0.234 |
| TNF-α, pg/mL† | - | - | - | 0.189 |
|  |  |  |  |  |
| Severity Scores |  |  |  |  |
| MELD score† | - | - | - | 0.145 |
| Child-Pugh score† | - | - | - | 0.198 |

*Mean ± SD; †Median (IQR); Bold indicates p<0.1 for univariate screening Abbreviations: ICU, intensive care unit; ACLF, acute-on-chronic liver failure; AKI, acute kidney injury; WBC, white blood cell; AST, aspartate aminotransferase; ALT, alanine aminotransferase; INR, international normalized ratio; EGF, epidermal growth factor; CRP, C-reactive protein; IL, interleukin; TNF, tumour necrosis factor; MELD, Model for End-Stage Liver Disease

| Supplementary Table 6: Internal validation using repeated stratified K-fold cross-validation | | | | | | |
| --- | --- | --- | --- | --- | --- | --- |
| *(10 repeats × 5 folds = 50 iterations per model)* | | | | | | |
|  |  |  |  |  |  |  |
| **Scoring System** | **Sample Size (n)** | **Events** | **Apparent AUC** | **CV-AUC (Mean)** | **95% CI** | **Optimism** |
| Part A: Infection | 60 | 19 | 0.759 | 0.749 | 0.354–1.000 | +0.009 |
| Part B: ICU Admission | 60 | 22 | 0.885 | 0.887 | 0.715–0.997 | -0.002 |
| Part C: Mortality | 69 | 27 | 0.815 | 0.815 | 0.568–0.977 | +0.000 |

AUC = Area Under the Receiver Operating Characteristic Curve

CV-AUC = Cross-validated AUC; CI = Confidence Interval (2.5th–97.5th percentile)

Optimism = Apparent AUC minus CV-AUC; positive values indicate potential overfitting

Complete case analysis was used for each model, meaning only patients with non-missing values for ALL predictors in that model were included. Note: Wide confidence intervals reflect inherent uncertainty with small sample sizes, not model instability

| Supplementary Table 7: Comparison of discrimination (AUC) between traditional scores and sick cirrhosis patient scores | | | | |
| --- | --- | --- | --- | --- |
|  |  |  |  |  |
| **Outcome** | **MELD3 AUC (95% CI)** | **CTP AUC (95% CI)** | **New Score AUC (95% CI)** | **Improvement vs MELD3** |
| Mortality (12-24 months) | 0.534 (0.404-0.658) | 0.553 (0.427-0.671) | 0.778 (0.661-0.886) | +0.244 |
| Infection at Hospitalization | 0.650 (0.500-0.782) | 0.629 (0.474-0.768) | 0.792 (0.677-0.894) | +0.142 |
| ICU Admission | 0.510 (0.375-0.640) | 0.581 (0.451-0.721) | 0.849 (0.760-0.925) | +0.338 |

AUC = Area Under the Receiver Operating Characteristic Curve; CI = Confidence Interval (bootstrap, 1000 iterations)

MELD3 = Model for End-Stage Liver Disease 3.0; CTP = Child-Turcotte-Pugh Score. New Score refers to the corresponding Sick Cirrhosis Patient Score (Part A for Infection, Part B for ICU, Part C for Mortality)
